# Atypical and delayed spinal cord MRI features of COVID-19-associated myelopathies: a report of four cases and literature review

**DOI:** 10.1007/s10072-024-07351-9

**Published:** 2024-03-02

**Authors:** Jie Wang, Huijun Zhang, Jingya Lin, Lingli Yang, Lipeng Zhao, Ailian Du

**Affiliations:** 1 Department of Neurology, Tongren Hospital, Shanghai Jiaotong University School of Medicine, Shanghai, 200336 China; 2 Institute of Neurology, Tongren Hospital, Shanghai Jiao Tong University School of Medicine, Shanghai, China

**Keywords:** COVID-19, Myelopathy, Magnetic resonance imaging, Atypical, Delay

## Abstract

We reported four patients with coronavirus disease 2019 (COVID-19)–associated myelopathies, highlighting the delayed and atypical spinal cord magnetic resonance imaging (MRI) features and the literature review. All four patients were males, aged 37 to 72 years old. The latencies from COVID-19 to the onset of myelitis were 5, 15, 30, and 80 days. The initial symptoms were numbness and weakness of lower limbs in three cases, and back pain with weakness of lower limbs in one case. The peak symptoms included paraplegia, sphincter dysfunction, sensory disturbance level, and spastic gait. The EDSS scores were 7.5, 9.0, 9.0, and 7.5, respectively. Magnetic resonance imaging (MRI) showed delayed atypical spinal cord lesions at onset, i.e., two cases without lesions, one with linear spinal meningeal enhancement, and one with punctate lesions on T2-weighted imaging (T2WI). During the follow-up period, punctate, linear, and cloudy lesions in the lateral and posterior funiculus were seen on T2WI in the peak stage. The prominent features of spinal cord lesions were linear spinal meningeal enhancement, the mismatch of deteriorated clinical symptoms, and inapparent MRI findings. All four patients were left with an obvious disability, with two patients completely bedridden and two who could stand with support. This report highlights the recognition of COVID-19-associated myelopathy even months after initial infection, especially in patients with delayed and atypical spinal cord findings on MRI.

## Background

Neurological manifestations after coronavirus disease 2019 (COVID-19) are severe clinical conditions, including encephalitis, myelitis, Guillain–Barre syndrome, and muscle diseases [1, 2]. Transverse myelitis (TM) after COVID-19 infection is not uncommon. The incidence of myelitis post COVID-19 infection was 0.5 per million based on the 1760 COVID-19 series from Italy [3], which may account for 1.2% of all neurological complications of COVID-19 [4, 5]. Notably, the clinical manifestations, evolution, and spinal cord magnetic resonance imaging (MRI) features of COVID-19-associated myelitis may differ significantly from those of traditional TM [6]. Some patients may present delayed onset for 3 months after infection [7]. Some presented typical TM of paraplegia and sphincter dysfunction with normal spinal cord MRI [8–10], which caused diagnosis confusion and potential treatment delay [7]. These findings suggested that normal spinal cord MRI presentation may be a predictor of poor prognosis [6, 9]. Therefore, improving the recognition of the clinical course and spinal cord MRI features of COVID-19-associated myelitis is of great significance. Herein, the clinical evolution (Table 1), atypical spinal cord MRI characteristics (Table 2), and treatment response of four patients with COVID-19-associated myelopathies were analyzed, and a literature review was presented (Table 3).

**Table 1:** Clinical features of the four cases

No	Sex	Age (y)	Severity of COVID-19	Latency (days)	Spastic gait	Limbs weakness	Sensory level	Sphincter dysfunction		Brisk reflex	Ankle clonus	Babinski sign	EDSS score
								Urinary retention	Constipation				
1	M	70	Mild	5	NS	+	T10	Partial	−	+	+	+	7.5
2	M	66	Mild	15	NS	+	T6	+	+	−	−	−	9
3	M	72	Asymptomatic	80	NS	+	T4	+	+	−	−	−	9
4	M	37	Mild	30	+	+	C4	−	+	+	+	+	7.5

*COVID-19* coronavirus disease 2019, *EDSS* Expanded Disability Status Scale, *Latency (days)* latency from symptoms of COVID-19 to onset of myelitis, *NS* not able to stand

**Table 2:** MRI of spinal cord of the four cases

No	Position	Initial	Repeated
Case 1	Thoracic (Fig. 1)	On day 13, sagittal T2 showed normal, with spots-like hyperintensity on axial T2. T1 + gadolinium scan showed intermittent meningeal enhancement, intramedullary cloudy-like enhancement on the sagittal plane, and patchy meningeal and intramedullary enhancement on the axial plane	On day 69 follow-up, sagittal T2 remained normal, and axial T2 showed scattered patchy high signals in lateral funiculus. No enhancement was observed after contrast
Case 2	Cervical (Fig. 2)	On day 53, T2 showed normal. T1 + gadolinium showed long-segmental spinal meningeal enhancement on the sagittal plane and scattered point enhancement on the axial plane	On day 63 when the symptoms worsened, T2 remained normal. Sagittal T1 + gadolinium showed weaker linear spinal meningeal enhancement, but new cloudy adjacent medulla enhancement. Axial T1 + gadolinium showed scattered point enhancement
Case 3	Cervico-thoracic (Fig. 3)	On day 10, sagittal T2 showed normal, and axial T2 showed point and patchy high signals	On day 14 when symptoms got worse, sagittal and axial T2 of the cervical spinal cord showed point and patchy high signals. Sagittal T1 + gadolinium showed long-segmental spinal meningeal enhancement. Axial T1 + gadolinium showed patchy enhancement in lateral funiculus
Case 4	Cervico-thoracic (Fig. 4)	On day 7, cervico-thoracic MRI in another hospital showed normal (data not acquired)	On day 54, sagittal T2 showed no significant abnormality in the cervical spinal cord, but long-segmental high signal in lower thoracic segments. Axial T2 showed symmetrical high signals in bilateral lateral funiculus of the cervical spinal cord and bilateral lateral and posterior funiculus of the thoracic spinal cord. Sagittal T1 + gadolinium showed long-segmental cervical spinal meningeal enhancement and linear thoracic spinal meningeal enhancement

**Table 3:** Clinical features of MRI-negative cases from literature review

No	Sex	Age (y)	Severity of COVID-19	Latency	Gait		Limbs weakness	Sensory level	Sphincter dysfunction			Brisk reflex	Ankle clonus	Babinski sign
					Spastic	Wide-based			Urinary retention	Urinary incontinence	Constipation			
1 [7]	F	65	Severe	9 weeks		+	+	T10	−	+	+	+	+	±
2 [8]	F	Early 30 s	Mild	2 weeks		+	−	T5–T6	+		+	+	NA	+
3 [9]	M	69	Asymptomatic	NA	NA		+	T8	+		+	+	NA	NA
4 [9]	M	64	Mild	3 to 4 months	Difficulty in tandem gait		+	Mid-thoracic	NA			+	NA	NA
5 [9]	F	64	Mild	5 to 6 months	+		+	−	NA			+	+	+
6 [9]	F	58	Moderate	3 weeks		+	+	T6	NA			+	NA	NA
7 [9]	M	63	Mild	2 months	+		−	NA	NA			+	NA	+
8 [10]	F	57	Mild	2 months	+		+	T7	+		+	+	+	+
9 [11]	M	50	Mild	1 day	NA		+	T6		+		NA	NA	±
10 [12]	M	63	Mild	12 days	NA		+	T10	NA			NA	NA	+

*COVID-19* coronavirus disease 2019, *Latency* latency from symptoms of COVID-19 to onset of myelitis, *NA* not available, *Sensory level* trunk sensory loss level

## Case presentation

The four patients were admitted to the Department of Neurology of Shanghai Tongren Hospital (Shanghai, China) from January 1 to March 30, 2023. All patients were assessed for clinical manifestations and differential diagnosis. This study was conducted in compliance with the 1964 Declaration of Helsinki. Informed consent, blood samples, and clinical evaluations were obtained from all participants, and the study was approved by the Ethic Committees of Shanghai Tongren Hospital (Ethic 2022–098-01).

### Case 1

**Fig. 1:**
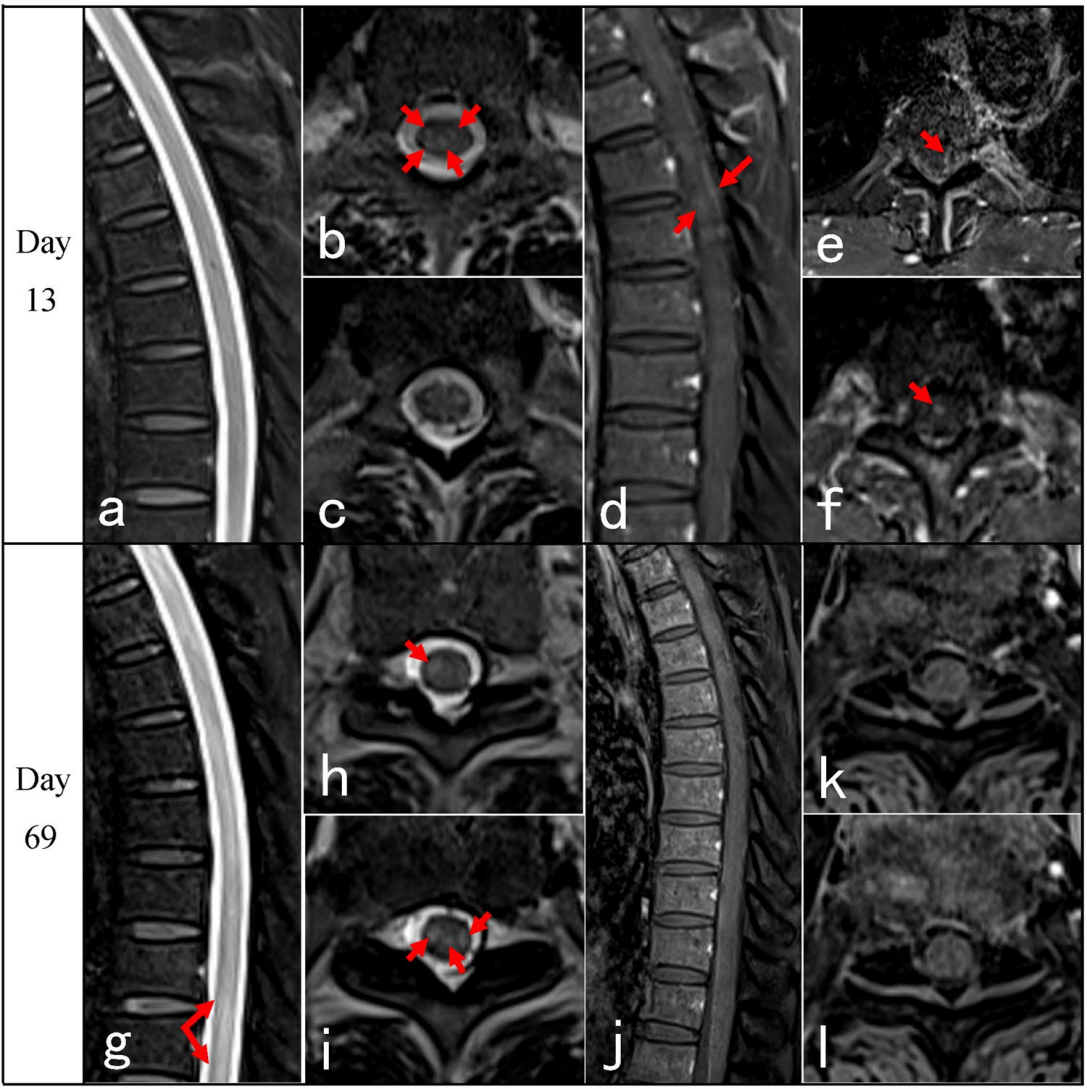
Thoracic spinal cord MRI of case 1: On day 13 from the onset, sagittal T2 did not show any abnormal signal (**a**). Axial T2 revealed spots-like hyperintensity (**b **and **c**, red arrows). Sagittal T1 + gadolinium showed intermittent meningeal enhancement and intramedullary cloudy-like enhancement (**d**, red arrows). Axial T1 + gadolinium showed scattered spots-like meningeal and intramedullary enhancement (**e**, **f**, red arrows). On day 69 follow-up, sagittal T2 showed longitudinal hyperintensities (**g**). Axial T2 showed scattered, patchy, intense signals in bilateral tract (**h**, **i**, red arrows). Sagittal and axial T1 + gadolinium showed no enhancement was observed on the bilateral tract lesions (**j**–**l**)

A 70-year-old male developed fever and fatigue by the end of December 2022, which lasted for 3 days. He tested positive twice for severe acute respiratory syndrome coronavirus-2 (SARS-CoV-2) antigen and was diagnosed as a mild case of COVID-19 according to the 9th Diagnosis and Treatment Protocol for COVID-19 [13]. After 5 days, he developed numbness and weakness in the right lower limb, which progressed to the left lower limb on the next day. The level of numbness ascended to the umbilicus on the 7th day, causing urinary incontinence. The patient was admitted to our hospital after 12 days. Neurological examination revealed that muscle strength was grade 2/5 on the left leg and 3 − /5 on the right leg based on the Medical Research Council (MRC) scale, bilateral hypermyotonia, brisk tendon reflexes, ankle clonus, and positive Babinski sign. There was bilateral sensory loss of pain and vibration below the level of the umbilicus. The laboratory analysis revealed a white blood cell count of 13.36 × 10^9^/L (4.0–10.0 × 10^9^/L), cerebrospinal fluid (CSF) cell count of 12 × 10^6^/L (0–10 × 10^6^/L), and elevated CSF protein of 0.71 g/L (< 0.45 g/L). Antibodies, including anti-aquaporins 4 (AQP-4), anti-myelin oligodendrocyte glycoprotein (MOG), and anti-glial fibrillary acidic protein (GFAP) in serum and CSF, were negative; also, oligoclonal band (OB) was negative. Thoracic MRI scan on day 13 was normal on sagittal T2, with spots-like hyperintensity on axial T2. T1 + gadolinium scan showed intermittent meningeal enhancement, intramedullary cloudy-like enhancement on the sagittal plane, and patchy meningeal and intramedullary enhancement on the axial plane. His brain MRI was normal. On day 69 follow-up, sagittal T2 remained normal, while axial T2 showed scattered patchy high signals in lateral funiculus. No enhancement was observed after contrast injection (Fig. 1). He received pulse intravenous methylprednisolone (PIM) of 500 mg/day from the 12th day of onset, followed by temporary improvement of lower limb weakness within the first 3 days, but was slightly aggravated from the 4th day. Subsequently, the patient was treated with plasma exchange (PLEX) from the 4th day but did not effectuate further improvement.

### Case 2

**Fig. 2:**
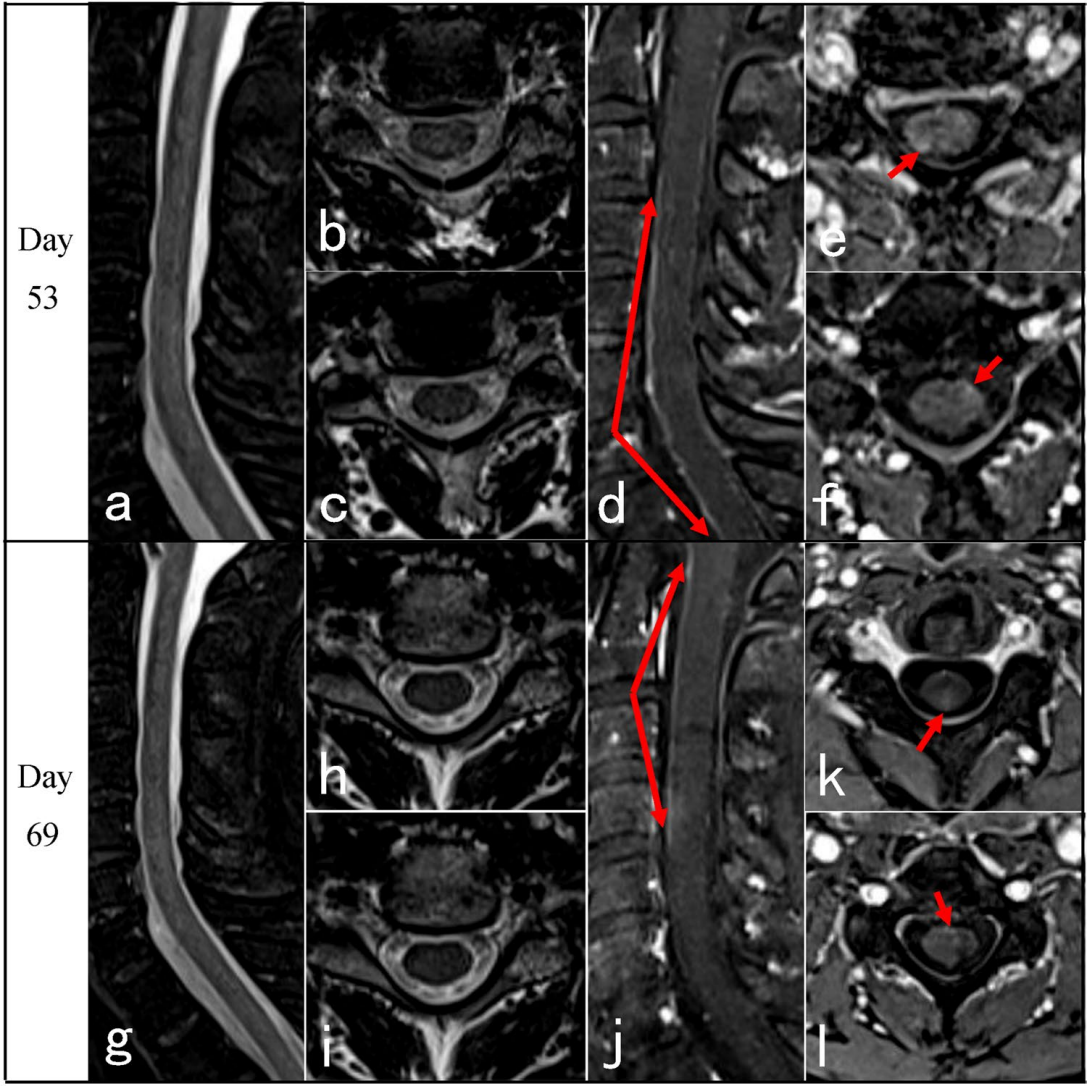
Cervical spinal cord MRI of case 2: On day 53 after the onset, T2-weighted MRI showed no significant abnormal signal (**a**, sagittal; **b**, **c**, axial). T1 + gadolinium showed long-segmental spinal meningeal enhancement (**d**, red arrows, sagittal) and scattered perispinal enhancement (**e**, **f**, red arrows, axial). But no intra-axial signal abnormality was observed. Despite the worsening of symptoms on day 63, sagittal and axial T2 showed no significant abnormal signal (**g**–**i**). Sagittal T1 + gadolinium showed that the linear spinal meningeal enhancement on day 53 weakened, while new cloudy enhancement was observed in the adjacent medulla (j, red arrows). Axial T1 + gadolinium showed scattered point enhancement (**k**, **l**, red arrows). The thoracic spinal cord MRI for this patient was normal (data not shown)

A 66-year-old male developed mild fever and cough in December 2022, which disappeared after 3 days. He tested positive twice for SARS-CoV-2 antigen and was diagnosed as a mild COVID-19 case. After 15 days, he presented sudden onset of back pain and heaviness in the back, and developed weakness in the bilateral lower limbs, which progressed to walking with support after 7 days and to complete immobility a month later. Numbness in the lower limbs started from the sole and progressed to the groins in about 1 week, followed by difficulty in urination. He visited the Emergency Department of our hospital for catheterization indwelling on the 49th day of disease onset and was hospitalized 2 days later. Neurological examination revealed that muscle strength was MRC grade 0/5 on the left lower limb and 2/5 on the right lower limb, with hypomyotonia, weakened tendon reflexes, and negative pyramidal signs. The sensory level was at the costal arches level, below which the pain and vibration sensation decreased bilaterally. After 3 days of PIM administration, he reported numbness in the hands, and the sensory level ascended to the level of the manubrium sterni. The laboratory analysis showed white blood cell count of 11.00 × 10^9^/L, CSF cell count of 1 × 10^6^/L, and CSF protein of 0.7 g/L. Anti-AQP4, anti-MOG, anti-GFAP, and anti-AQP1 antibodies in serum and CSF were negative; also, OB was negative. Cervical MRI scan on day 53 after the onset was normal on T2-weighted scan. T1 + gadolinium showed long-segmental spinal meningeal enhancement on the sagittal plane and scattered point enhancement on the axial plane. When the symptoms worsened on day 63, T2-weighted scan remained normal. Sagittal T1 + gadolinium showed weak linear spinal meningeal enhancement, but new cloudy enhancement was observed in the adjacent medulla. Axial T1 + gadolinium showed scattered point enhancement (Figure 2). Brain MRI at the same time was normal. The patient received PIM of 500 mg/day from the 54th day with a slight improvement in the weakness of the lower limbs in the first 3 days but worsened with ascending weakness and dysesthesia level to the right arm on the 4th day. Additional PLEX treatment was performed, but no improvement was detected.

### Case 3

**Fig. 3:**
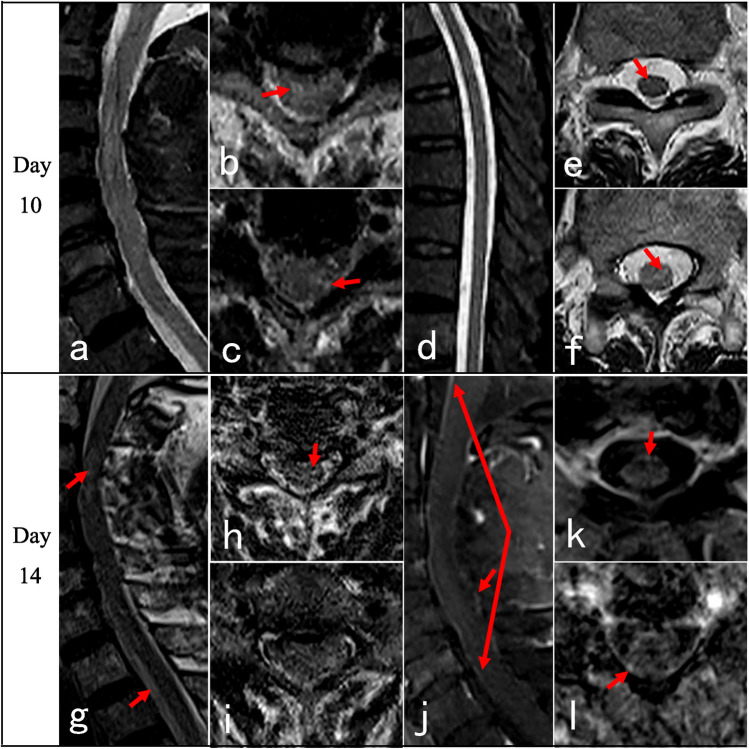
Cervico-thoracic spinal cord MRI of case 3: On day 10 after the onset, cervico (**a**) and thoracic (**d**) sagittal T2 scan did not show any significant abnormal signal. Axial T2 showed scattered points and patchy high signals of the cervico (**b**, **c**, red arrows) and thoracic spinal cord (**e**, **f**, red arrows). On day 14 when the symptoms worsened, sagittal and axial T2 of the cervical spinal cord showed point and patchy high signals (**g**–**i**, red arrows). Sagittal T1 + gadolinium showed long-segmental spinal meningeal enhancement (**j**, red arrows). Axial T1 + gadolinium showed patchy enhancement in lateral cord (**k **and **l**, red arrows)

A 72-year-old male tested positive for SARS-CoV-2 antigen without symptoms for three consecutive days in mid-December 2022. He was diagnosed as an asymptomatic COVID-19 case. After 80 days, he developed slight numbness in the hands and feet and weakness in lower limbs. On the 9th day, he developed urinary retention needing urethral catheterization. On the 10th day, the weakness progressed rapidly, and the patient was admitted to our hospital. Neurological examination revealed that muscle strength was MRC grade 5 − /5 in the right upper limb and left hand, 2 + /5 in the right lower limb, and 3 − /5 in the left lower limb, with decreased tendon reflexes and negative pyramidal signs. Dysesthesia level was at T4 level. After 2 days of PIM (500 mg/day), his muscle strength worsened to MRC grade 0/5 in the bilateral lower limbs, 2 − /5 in the right upper limb, and 4/5 in the left upper limb; then, PIM was ceased. Laboratory analysis showed CSF cell count of 1 × 10^6^/L, CSF protein of 0.6 g/L, and negative anti-AQP4, anti-MOG, anti-GFAP, and anti-AQP1 antibodies in serum and CSF. T2-weighted scan of cervico-thoracic MRI on day 10 was normal on the sagittal plane, while point and patchy high signals were detected on the axial plane. On day 14 when the symptoms got worse, sagittal and axial T2-weighted scan of the cervical spinal cord showed point and patchy high signals. Sagittal T1 + gadolinium showed long-segmental spinal meningeal enhancement, while axial T1 + gadolinium showed patchy enhancement in lateral funiculus (Fig. 3). Brain MRI was normal at the same time.

### Case 4

**Fig. 4:**
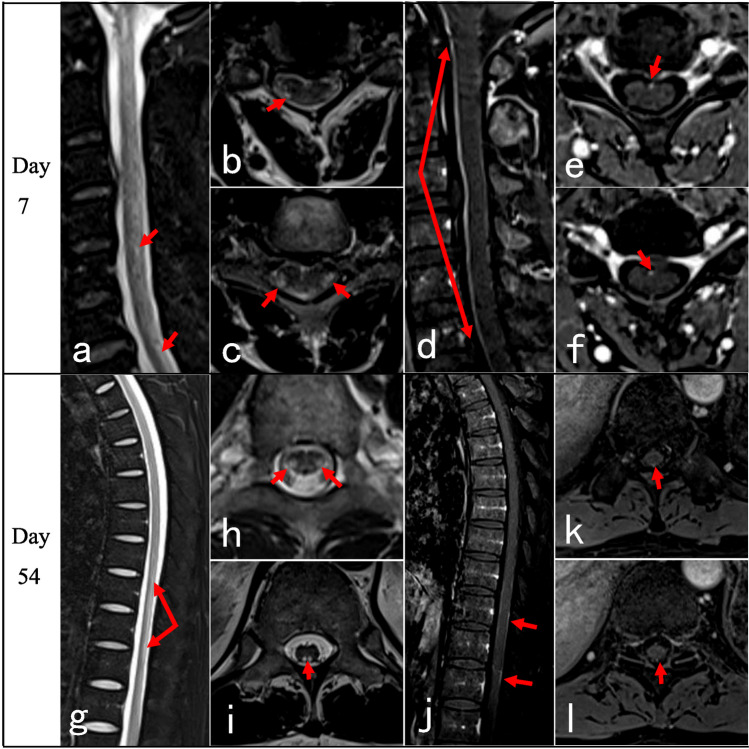
Cervico-thoracic spinal cord MRI of case 4: On day 7 after the onset, sagittal T2 scan of the cervical spinal cord showed scattered abnormal signals (**a**). Axial T2 of the cervical spinal cord showed symmetrical high signals in bilateral lateral cords (**b**, **c**, red arrows). Sagittal T1 + gadolinium showed long-segmental spinal meningeal enhancement (**d**–**f**, red arrows). On day 54 after the onset, T2 of the thoracic spinal cord showed long-segmental intense signal in the lower thoracic segments on the sagittal section (**g**, red arrows) and symmetrical high signals in the bilateral lateral and posterior cords on the axial section (**h**, **i**, arrows). Weak linear spinal meningeal enhancement was observed after contrast injection (**j**–**l**, arrows)

A 37-year-old male developed cough, fatigue, and chilliness, which lasted for 3 days in late December 2022. He tested positive for SARS-CoV-2 antigen and was diagnosed as a mild COVID-19 case. After 30 days, he developed numbness and weakness in the bilateral lower limbs, dragging step, and abnormal gait. The symptoms progressed in addition to constipation and the sensation of stepping on cotton on the 54th day when he was admitted to our hospital. Neurological examination revealed that muscle strength was MRC grade 5 − /5 in the left hand, 4/5 at the proximal lower limbs, and 5 − /5 at the distal lower limbs, and hypermyotonia, spastic gait, brisk tendon reflex, bilateral positive Hoffman’s sign and ankle clonus, and positive Babinski’s sign. The sensation of pinprick and vibration decreased bilaterally below the level of C4. Laboratory analysis showed that CSF cell count was 4 × 10^6^/L, CSF protein was 0.45 g/L, and anti-AQP4, anti-MOG, anti-GFAP, and anti-AQP1 antibodies were negative in serum and CSF; also, OB was negative. Cervico spinal cord MRI scan showed scattered high signals on sagittal T2 section and symmetrical high signals in bilateral lateral cord on axial T2 section. Sagittal T1 + gadolinium showed long-segmental cervical spinal meningeal enhancement. Long-segmental high signal was detected in the lower thoracic segments and symmetrical high signals on bilateral lateral and posterior funiculus. Sagittal T1 + gadolinium showed weak linear thoracic spinal meningeal enhancement (Fig. 4). Brain MRI obtained at the 54th day was normal. The patient received intravenous methylprednisolone of 40 mg/day from day 56 of the onset for 3 days, which was terminated due to the worsening sensation of stepping on cotton.

## Discussion

SARS-CoV-2 can affect the nervous system through direct and indirect mechanisms, such as mediating immune response and causing hypercoagulable states [14], causing various clinical symptoms. The neurological manifestations include headache and dizziness, myalgia and fatigue, stroke, encephalitis and myelitis, encephalopathy syndrome, and peripheral neuropathy. And myelitis post-SARS-CoV-2 infection accounts for 1.2% of the neurological associations [6, 15]. TM is defined as acute or subacute spinal cord dysfunction with motor, sensory, and autonomic dysfunction below the lesion level [16]. The etiology includes demyelination, inflammation, paraneoplasia, and infection [17].

Currently, there are no established diagnostic criteria for COVID-19-associated myelitis. Ellul et al. [2] proposed three diagnostic levels of SARS-CoV-2-associated myelitis/myelopathies: confirmed, probable, and possible. Based on this suggestion, all four patients suffered from COVID-19 during the pandemic in China. They were diagnosed as mild or asymptomatic cases of COVID-19 according to the 9th Edition of Diagnostic and Treatment Criteria Proposed by the National Health Care Commission [13]. Moreover, they presented similar neurological manifestations, including weakness in the bilateral lower limbs, sensory level of the trunk, sphincter dysfunction, and pyramidal signs that could not be explained by other causes. MRI of the spinal cord exhibited no extra medullary compressions nor spinal cord arteriovenous malformations. According to the criteria suggested by Ellul et al. [2], the cases were diagnosed as COVID-19-associated myelopathy. In addition, metabolic myelopathy such as vitamin B_12_ or copper deficiencies has been excluded by normal blood count, normal vitamin level, and normal serum homocysteine. And other immune myelitis was excluded by testing for relevant antibodies, such as AQP4, MOG, GFAP, and serum vitamins for all four patients.

The spinal cord MRI of COVID-19-associated myelitis is mostly classical T2 hyperintensities affecting localized or longitudinal spinal cord segments [4–6], while atypical MRI features have been described as discontinuous, pointy or patchy, and non-transverse lesions. Schulte et al. [5] reviewed the spinal cord lesions on MRI of 15 patients with COVID-19-associated myelitis. The study demonstrated classic long-segmental lesions extended to three or more spinal cord segments in 93.3% of individuals. T2 hyperintensities were described as “patchy lesions” in three cases and “patchy enhancement” in two cases. Adamec et al. [6] reported discontinued T2 hyperintense lesions in 7/13 patients. Memon et al. [7] described multifocal T2 signal abnormality on sagittal STIR, which was mostly distributed in the lateral funiculus on axial T2 of the repeated MRI. Linear meningeal enhancement on an axial gadolinium T1-weighted scan might be a major characteristic for COVID-19-associated myelitis, observed in our four cases. The before and after self-contrast injection of a single patient (case 2) follow-up exhibited a dynamic change in the spinal cord lesion. The initial MRI on the 53rd day of onset showed long-segmental spinal meningeal enhancement. Repeat-contrast MRI on the 63rd day with worsened symptoms displayed weak meningeal enhancement, while intramedullary cloudy enhancement appeared adjacent to meningeal enhancement. This finding indicated that the lesions of COVID-19-associated myelitis develop gradually from the spinal meningeal to the spinal cord parenchyma. The finding of linear meningeal enhancement is valuable for the early identification of COVID-19-associated myelitis.

Typical manifestations, including gate disturbance, limb weakness, sensory level, and sphincter dysfunction, in COVID-19-associated myelitis have been described as transverse myelitis [5]. In order to understand the clinical characteristics of COVID-19-associated myelitis, we reviewed the literature on COVID-19-associated myelitis with atypical and negative MRI images and identified 10 cases of MRI-negative COVID-19-associated myelitis. The clinical characteristics of these patients are presented in Table 3. Five/10 patients are female, and the age of the cohort was early 30 s to 69 years. The severity of COVID-19 infection was asymptomatic in one case, moderate in one case, mild in seven cases, and severe in one case. The latency from COVID-19 to onset of myelitis ranged from 1 day to 5–6 months. The patients presented gait disturbances, including spastic gait (3/10), wide-based gait (3/10), and difficulty in tandem gait (1/10), limb weakness (8/10), and trunk sensory loss level at thoracic planes (8/10). Sphincter dysfunction included urinary retention (3/10), urinary incontinence (2/10), constipation (4/10), brisk reflex (8/10), ankle clonus (3/10), positive Babinski sign (5/10), and equivocal Babinski sign (2/10). As for case 3, he had a latency of 80 days after antigen positive for three times. We still diagnosed him as COVID-19-associated myelopathy because he had typical symptoms and identical spinal cord MRI lesions to the other three patients. And also, long latency is common in other related reports [6, 7]. In the retrospective analysis by Adamec et al. [6], 4/12 patients with initial normal spinal cord MRI showed lesions when repeated after 15 to 44 days, suggesting that these lesions on MRI of COVID-19-associated myelitis have a time lag (a latency from symptom of myelopathy to spinal cord MRI lesions). We also observed time lag in the present study; none of the four patients showed any obvious abnormality on the first MRI at the onset but showed atypical lesions on repeated MRI. Thus, for the patient with the first negative MRI, a repeat MRI scan is necessary.

The mechanism of initial negative MRI findings of COVID-19-associated myelitis was not clarified. Memon et al. [7] reported a late-onset patient with a normal initial MRI showing abnormal signals on the repeat MRI 4 months later. The study speculated an intense axonal degeneration without demyelination after severe viral-induced pathogenesis, which did not appear on the MRI scan in the previous disease course. COVID-19-associated myelitis with normal early MRI scan suggested pathological mechanisms of this disease that differed from traditional immune-mediated myelitis. Abrams et al. [9] speculated that SARS-CoV-2 infection causes micro-hemorrhages and micro-thromboses of small vessels, resulting in small vessel stroke of the spinal cord. This might explain the lack of apparent spinal cord MRI findings. Some studies suggested that a normal MRI of the spinal cord may be a sign of poor prognosis [6, 9]. The four patients in this study did not benefit from pulse therapy of methylprednisolone and PLEX. Adamec et al. [6] showed that > 80% of patients received a combination of corticosteroids and PLEX or intravenous immunoglobulins (IVIG), and only 25.4–30.8% of patients had a complete or significant recovery, while 12.4–19% showed no recovery, and 9.5% had a fatal outcome. The findings suggested that normal MRI is an independent predictor for poor outcomes.

In conclusion, we reported four cases of COVID-19-associated myelitis with delayed and atypical spinal cord MRI characteristics. Atypical MRI manifestations include discontinuous, punctate, or scattered intramedullary lesions, with linear spinal meningeal enhancement or cloudy intramedullary enhancement. The study on the mechanism of atypical or negative early MRI findings and the reason for poor immunotherapy effect would guide early diagnosis and help design appropriate therapeutic strategies for COVID-19-associated myelitis.

## References

[1] Guan WJ, Ni ZY, Hu Y (2020). Clinical characteristics of coronavirus disease 2019 in China. N Engl J Med.

[2] Ellul MA, Benjamin L, Singh B (2020). Neurological associations of COVID-19. Lancet Neurol.

[3] Rifino N, Censori B, Agazzi E (2021). Neurologic manifestations in 1760 COVID-19 patients admitted to Papa Giovanni XXIII Hospital, Bergamo, Italy. J Neurol.

[4] Román GC, Gracia F, Torres A (2021). Acute transverse myelitis (ATM): clinical review of 43 patients with COVID-19-associated ATM and 3 post-vaccination ATM serious adverse events with the ChAdOx1 nCoV-19 vaccine (AZD1222). Front Immunol.

[5] Schulte EC, Hauer L, Kunz AB (2021). Systematic review of cases of acute myelitis in individuals with COVID-19. Eur J Neurol.

[6] Adamec I, Brecl Jakob G, Drulović J (2022). Transverse myelitis following COVID-19: Insights from a multi-center study and systematic literature review. J Neurol Sci.

[7] Memon AB, Al-Hader R, Patel S (2021). Late-onset rapidly progressive MRI- negative-myelitis after COVID-19 illness. Clin Neurol Neurosurg.

[8] Masaad D, Youssef S, Safadi MF (2022). MRI-negative myelitis associated with cerebral venous thrombosis after COVID-19 infection. BMJ Case Rep.

[9] Abrams R, Safavi F, Tuhrim S (2021). MRI negative myelopathy post mild SARS-CoV-2 infection: vasculopathy or inflammatory myelitis. J Neurovirol.

[10] Zukic S, Topcic E, Hodzic R (2022). Spastic paraparesis after SARS-CoV-2 infection without radiological changes. Cureus.

[11] Águila-Gordo D, Manuel Flores-Barragán J, Ferragut-Lloret F (2020). Acute myelitis and SARS-CoV-2 infection. A new etiology of myelitis. J Clin Neurosci.

[12] Zachariadis A, Tulbu A, Strambo D (2020). Transverse myelitis related to COVID-19 infection. J Neurol.

[13] National Health Commission of the People’s Republic of China (2022). Diagnosis and treatment protocol for COVID-19 (ninth trial edition). Chin J Clin Infect Dis.

[14] Czarnowska A, Zajkowska J, Kułakowska A (2023). Impact of SARS-CoV-2 on the nervous system. Neurol Neurochir Pol.

[15] Branch CMDAN (2023). Expert recommendations for clinical treatment of neurological diseases caused by SARS-CoV-2 infection. PLA Med J.

[16] Transverse Myelitis Consortium Working Group (2002). Proposed diagnostic criteria and nosology of acute transverse myelitis. Neurology.

[17] West TW, Hess C, Cree BA (2012). Acute transverse myelitis: demyelinating, inflammatory, and infectious myelopathies. Semin Neurol.

